# Assessing fidelity of cognitive behavioral therapy in rural VA clinics: design of a randomized implementation effectiveness (hybrid type III) trial

**DOI:** 10.1186/s13012-016-0432-4

**Published:** 2016-05-10

**Authors:** Michael A. Cucciare, Geoffrey M. Curran, Michelle G. Craske, Traci Abraham, Michael B. McCarthur, Kathy Marchant-Miros, Jan A. Lindsay, Michael R. Kauth, Sara J. Landes, Greer Sullivan

**Affiliations:** 1 Center for Mental Healthcare and Outcomes Research, Central Arkansas Veterans Affairs Healthcare System, 2200 Fort Roots Drive, North Little Rock, AR 72114 USA; 2 Department of Psychiatry, University of Arkansas for Medical Sciences, Little Rock, AR USA; 3 VA South Central Mental Illness Research Education, and Clinical Center, North Little Rock, AR USA; 4 VA South Central Mental Illness Research Education, and Clinical Center, Houston, TX USA; 5 Department of Pharmacy Practice, College of Pharmacy, University of Arkansas for Medical Sciences, Little Rock, AR USA; 6 Department of Psychology, University of California, Los Angeles, CA USA; 7 Center for Innovations in Quality, Effectiveness and Safety, Michael E. Debakey VA Medical Center, Houston, TX USA; 8 Center for Healthy Communities, Department of Social Medicine and Population Medicine, School of Medicine, University of California, Riverside, Riverside, California USA; 9 Department of Psychiatry, Division of Clinical Sciences, University of California, Riverside, CA USA

**Keywords:** Cognitive behavioral therapy, Treatment fidelity, Veterans, Evidence-based practices, Rural

## Abstract

**Background:**

Broadly disseminating and implementing evidence-based psychotherapies with high fidelity, particularly cognitive behavioral therapy (CBT), has proved challenging for many health-care systems, including the Department of Veterans Affairs, especially in primary care settings such as small or remote clinics. A computer-based tool (based on the coordinated anxiety learning and management (CALM) program) was designed to support primary care-based mental health providers in delivering CBT. The objectives of this study are to modify the CALM tool to meet the needs of mental health clinicians in veterans affairs (VA) community-based outpatient clinics (CBOCs) and rural "veterans", use external facilitation to implement CBT and determine the effect of the CALM tool versus a manualized version of CALM to improve fidelity to the CBT treatment model, and conduct a needs assessment to understand how best to support future implementation of the CALM tool in routine care.

**Methods/design:**

Focus groups will inform the redesign of the CALM tool. Mental health providers at regional VA CBOCs; CBT experts; VA experts in implementation of evidence-based mental health practices; and veterans with generalized anxiety disorder, panic disorder, social anxiety disorder, posttraumatic stress disorder, "with or without" depression will be recruited. A hybrid type III design will be used to examine the effect of receiving CBT training plus either the CALM tool or a manual version of CALM on treatment fidelity. External facilitation will be used as the overarching strategy to implement both CBT delivery methods. Data will also be collected on symptoms of the targeted disorders. To help prepare for the future implementation of the CALM tool in VA CBOCs, we will perform an implementation need assessment with mental health providers participating in the clinical trial and their CBOC directors.

**Discussion:**

This project will help inform strategies for delivering CBT with high fidelity in VA CBOCs to veterans with anxiety disorders and PTSD with or without depression. If successful, results of this study could be used to inform a national rollout of the CALM tool in VA CBOCs including providing recommendations for optimizing the adoption and sustained use of the computerized CALM tool among mental health providers in this setting.

**Trial registration:**

ClinicalTrials.gov, NCT02488551

## Background

Multiple randomized controlled trials (RCTs) have demonstrated that evidence-based psychotherapies (EBPs), particularly cognitive behavioral therapy (CBT) [1, 2], are highly effective for treating anxiety and depression, the most common mental health disorders in primary care settings [3–5]. The Department of Veterans Affairs (VA) has made it a national care priority to increase the availability and accessibility of EBPs across all VA medical facilities and clinics, to Veterans needing mental health care [6].

However, broadly disseminating and implementing (a process often referred to as “scaling up”) these and other EBPs has been a challenge in many health-care systems [7], including the VA [8, 9]. While training of clinicians is needed for scaling up, training alone is inadequate to assure that EBPs are not only implemented but also implemented with enough fidelity to optimize their clinical effectiveness [10]. Without adequate implementation and fidelity, patients realize few benefits and the resources devoted to training clinicians fail to improve patient outcomes.

Implementing EBPs in small or remote primary care clinics, such as many VA community-based outpatient clinics (CBOCs), can be especially challenging. A 2008 study showed that only 22 % of veterans with depression, anxiety, or posttraumatic stress disorder (PTSD) received at least one session of psychotherapy and rural veterans were even less likely than urban veterans to receive any psychotherapy [11]. Furthermore, the quality of the psychotherapy received in these settings is unknown. Mental health providers in CBOCs, many of which are located in rural and highly rural areas, are often the only mental health provider at the clinic and may be isolated from their peers and from educational resources. Further, some of these providers have been trained in specific EBPs [6] but may not have the resources or time needed to become proficient in these skills. Therefore, efforts to implement tools to support and assist CBOC mental health providers in delivering EBPs with high fidelity are needed.

The National Institute of Mental Health-Funded Coordinated Anxiety Learning and Management (CALM) study [12–14] faced a similar challenge in implementing CBT in 17 non-VA primary care clinics across the USA with providers, usually nurses and social workers, most of whom had no previous training or experience in CBT. To meet this challenge, researchers developed a computer-based tool to support primary care-based mental health providers in delivering CBT to patients with a range of disorders, including panic disorder (PD), generalized anxiety disorder (GAD), social anxiety disorder (SAD), PTSD, and comorbid depression [15].

### CALM program

The CALM program, including the computer and manual versions, was created to guide and train mental health providers in using CBT. The computerized CALM tool was not intended, like some other computer-based psychotherapy tools [16, 17], as a self-help intervention (i.e., patient facing without provider involvement). The CALM tool involves the patient and provider looking at the computer screen together and proceeding through the modules at their own pace [15]. Continuous use of this tool in treatment may have the added benefit of helping to maintain fidelity to the CBT treatment model. The CALM CBT tool is unique in that it can be used to treat anxiety disorders (PD, GAD, SAD), PTSD, and comorbid depression. This is accomplished through the use of basic CBT modules that are commonly employed across these disorders plus additional, branching modules that are disorder-specific [13]. In one study, the CALM tool, used by 13 providers in treating more than 250 patients in a variety of primary care clinics, was found to be acceptable to providers and patients and resulted in substantial treatment engagement and homework compliance [15]. Most importantly, the CALM tool is clinically effective (relative to standard care) [12] for all disorders (PTSD, PD, GAD, SAD with or without depression) [13]. Furthermore, the improved clinical outcomes achieved in CALM appear to be due primarily to receipt of CBT delivered with this computer program [12]. A tool providing fidelity support to mental health providers who are social workers, nurses, masters’ level counselors, and psychologists in primary care could help support VA CBOCs providers in delivering CBT and also improve outcomes.

### Implementation strategies and study aims

Implementing CBT in large health-care systems such as the VA is a substantial challenge [18] as barriers to implementation tend to vary at the site and provider levels. Initial training in EBPs typically is not sufficient for promoting its sustained use, with researchers suggesting a need for more intensive facilitation to address barriers to promote the use of a new practice in a “real-world” setting [18]. Facilitation is a key component of the promoting action on research implementation in health services framework, which specifies the potential roles of a facilitator in promoting the adoption of new clinical practices [19, 20]. For example, external facilitation can be used to address site- and provider-specific implementation barriers by using a facilitator who understands the complexities of the specific health-care system and who works with clinical teams to address implementation challenges as they arise [10, 21–23]. Facilitators use problem-solving, provide interpersonal support, and function as mentors to clinical teams to support the adoption and sustained use of a new practice [21]. In one study, external facilitation was used in ten VA clinics to implement CBT and was associated with a larger increase (19 %) in the use of CBT from baseline when compared to ten VA clinics (4 %) not receiving facilitation [21]. Together, these findings suggest that external facilitation is a key component to promoting the adoption of EBPs, and especially in large health-care settings where barriers to implementation can be numerous and vary across clinics and providers.

### Current study

The goal of this article is to describe our study, which involves four aims: to (a) modify the CALM tool to meet the needs of CBOC mental health providers and rural veterans; (b) use external facilitation to implement two CBT delivery methods (CALM tool versus CALM manual); (c) determine the effects of these two delivery methods to improve fidelity to the CBT treatment model; and (d) conduct a needs assessment to understand how best to support the future implementation of the CALM tool in routine VA care. A hybrid type III study design was chosen to allow for a primary focus on comparing two implementation strategies or delivery methods (CALM tool or CALM manual) of CBT and also collect patient-level outcome data. We will examine the effect of these tool delivery methods on CBT treatment fidelity (primary outcome). External facilitation will be used as an overarching implementation strategy to support the adoption of both methods for delivering CBT. Our secondary outcomes include patient-level data on reductions in anxiety and depressive symptoms among veteran patients over time in both conditions. Collecting patient-level outcomes will fill an important gap in CALM treatment outcome literature by providing data on the clinical effectiveness of CALM for veteran patients receiving care in VA CBOCs.

## Methods/design

The following section(s) describe the three study aims and hypotheses, study participants, data collection, and analysis plans that will be used to achieve each aim. Human subject approval for aim 1 of this study was granted by the Central Arkansas Veterans Healthcare System Research Committee. Aims 2 and 3 were approved by the Veterans Affairs Central Institutional Review Board.

### Aim 1: modify the CALM tool

We used methods common to the field of instructional design and technology to guide the redesign of the CALM computer tool for use in VA CBOCs. Instructional design and technology is a field of study that provides a systematic process for applying human and media resources to efficiently accomplish instructional goals [24]. The wide variety of instructional design and technology models described in the literature [25–27] share the core elements of analysis, design, development, implementation, and evaluation. These core elements are applied in an iterative and formative process. While these methods were originally designed to develop instructional materials, they are well suited for developing and refining online therapeutic-didactic tools and are consistent with formative evaluation methods commonly used in implementation research [10, 28].

*Analysis* typically includes inquiry about the audience or end user, the potential goals of the training/program, and the context in which the training/program will occur. *Design* (or *redesign* in the present study) is typically concerned with identifying goals and objectives for content (e.g., changing some treatment vignettes to reflect veterans and their perspectives and requirements for reading level) and a plan for “look and feel” (e.g., web presentation, toolbars, color scheme). *Development* refers to the construction of the prototype materials (e.g., the web tool with videos, all navigation working). *Implementation* refers to the process of putting the product into practical effect, i.e., when the content is posted to the web and the target audience uses it. *Evaluation* is seen as a continuous formative activity throughout the process. We hypothesize that the modified CALM tool will be acceptable to CBOC mental health providers and veterans.

### Study participants

We aim to collect qualitative data from four stakeholder groups: (a) CBOC mental health providers; (b) veterans who have an anxiety disorder or PTSD with or without depression.; (c) expert CBT clinicians; and (d) experts in the implementation of EBPs within the VA (Table 1). Feedback from CBOC mental health providers will help ensure that the content is presented in a way that is acceptable to them, that clinical vignettes and case studies presented are realistic, and that the navigation and flow of the material meets their clinical needs. Providers will be recruited from CBOCs located in the south central VA region of care encompassing Oklahoma and east of Texas to the Florida panhandle and Arkansas to the Gulf of Mexico. Feedback from veterans, recruited from this same region of CBOCs, will help ensure that the CBT material is acceptable to the target audience of patients; the case examples are reflective of their experience and that homework assignments are understandable and feasible to complete. Feedback from CBT experts, recruited from VA and non-VA locations, will help ensure that the empirical support underlying the tool is not compromised. Feedback from VA implementation experts will help the research team create the facilitation strategies to be used at the CBOCs. These strategies will support implementation of both the CBT delivery methods, as both will need facilitation to support training of the providers and their participation in continuing consultation for CBT.

**Table 1:** Participant eligibility criteria for each study aim

Study aim	Participants	Eligibility criteria
Aim 1: modify the CALM tool	Mental health providers (*n* = 8)	Inclusion criteria -works in a VISN 16 CBOC
	Veterans (*n* = 8)	Inclusion criteria -diagnosed with GAD, PD, SAD, and PTSD with or without depression within the last year -had at least two mental health visits at a VISN 16 CBOC in the prior year
	CBT experts (*n* = 4)	Inclusion criteria -VA or non-VA are eligible
	- Experts in the implementation of EBPs within VA (*n* = 4)	Inclusion criteria -VA employee involved in national “rollouts” of EBPs
Aim 2: hybrid type III effectiveness-implementation study	- Mental health providers (*n* = 34)	Inclusion criteria: -willing to receive a 3-day CBT training -has an office with a computer -willing to be audiotaped and participate in clinical supervision -willing to be randomized to CALM tool or manual version of CALM
	Veterans (*n* = 340)	Inclusion criteria: -patient of a participating provider -plan to continue to receive mental health care -diagnosed with PD, SAD, GAD, or PTSD with or without depression -want to receive CBT -willing to have therapy sessions audiotaped -willing to participate in clinical assessments Exclusion criteria: -no evidence of a significant cognitive impairment -not actively suicidal -no comorbid substance use disorders, schizophrenia, or bipolar disorder -not completed a course of CBT or cognitive processing therapy
Aim 3: implementation needs assessment	Mental health providers (*n* = 34)	Inclusion criteria: -must have participated in aim 2 -CBOCs with mental health providers participating in aim 2
	- CBOC directors	

### Data collection

All data will be collected in focus groups. Focus groups are a standard method in “new product development” and are widely used by marketing researchers to get feedback on new and existing products [29]. Focus groups provide a flexible mechanism for gaining insight into how others think, and they provide an efficient mechanism for understanding a range of diverse opinions at a meaningful level of detail [30]. One focus group will be created for each of the four stakeholder groups. Each focus group will meet at least three times to provide iterative feedback on the redesign of the CALM tool (via the analysis, design, development, implementation, and evaluation model). The first focus group will review the existing CALM tool and provide recommendations on revisions and new elements, the second will review the initial design plans and storyboards (“mock ups”) for the revisions and new elements, and the third focus group will review the prototype tool in a live web demonstration with a test clinician and test patient. The third focus group will have the opportunity to observe the CALM tool operating exactly as it will during the study trial.

The focus groups for the stakeholder groups (except veterans) will occur by teleconference with the assistance of Lync, an online meeting portal that allows participants to view the same material online simultaneously. Participants are able to leave written comments and feedback in the “chat room” as well as provide verbal feedback on the phone. The veteran focus groups will be in-person to limit potential barriers (access to teleconference software) to participating. Focus groups will be conducted by two individuals—one with expertise in the CALM tool and one with expertise in eliciting qualitative feedback. The group leaders will use interview guides that are helpful in keeping the interviews “on track” and minimizing the possibility that the interviews will be completed without “covering” topics that the study team feels are important to discuss [31]. Use of a guide, however, will not limit the interviewers from asking new questions or following participants along new lines of inquiry [32].

### Data analysis

To quickly produce the information needed to revise the CALM tool, we will use rapid data analytic techniques informed by Sobo et al. [33, 34]. Two notetakers will listen to audio recordings of the focus groups (and document recommended modifications provided in the chat room function of the online meeting portal) and summarize the feedback and recommendations from each focus group. They will resolve any differences that might have arisen from their separate note-taking. The notetakers will present their summaries to the focus group leaders and collectively finalize the feedback summaries from each group. The feedback summaries from each focus group will be used to develop a document listing categories (e.g., content, look and feel) that will be used to revise the CALM tool. For example, the first set of summaries will be provided after the focus groups view and respond to the current tool. A second set of summaries (or more if needed) will be provided after the focus groups view and respond to the design plans and draft storyboards of revisions. A third set of summaries (or more if needed) will be provided after the prototype has been reviewed and tested.

### Prototype development

The research team and the instructional/web design team that developed the original version of the CALM tool will work together to develop the redesigned version of the CALM computer tool. Possible modifications may include revising the language used to present the material, updating the graphics and images, and developing new instructional video clips with actors portraying veteran patients and veteran-specific scenarios.

### Aim 2: hybrid type III study

Our second aim we will be to use a hybrid type III [35] study design to conduct an RCT comparing the effect of a modified CALM tool versus that of a manualized version of CALM on our primary outcome—CBOC mental health provider fidelity to CBT. A hybrid III study design was used given (a) a substantial and clear evidence base indicating improved clinical outcomes associated with patients receiving CALM; (b) a strong need to identify optimal delivery methods for implementing CBT in VA CBOCs; and (c) the fact that CALM has significant “implementation momentum” [35] as our VA Central Office research partners are interested in adapting the CALM tool for use in VA CBOCs. Mental health providers will be randomized to receive CBT training plus the CALM tool or a manualized version of CALM. We hypothesize that providers randomized to receive CBT training and the CALM tool will demonstrate higher treatment fidelity than providers receiving the manual. We also hypothesize that veteran patients of providers randomized to receive CBT training and the CALM tool will have clinical outcomes superior to veteran patients of providers who receive the manual.

### Study participants

Participants will include mental health providers and veteran patients. We will recruit 34 mental health providers (all disciplines eligible) to receive training in CBT. Upon completion of training, each mental health provider will invite ten of his/her patients to participate in CBT treatment (Table 1).

### External facilitation

Our experience and the literature have shown that coupling training with a site implementation strategy optimizes the benefits of EBP training. External facilitation increases providers’ success in bringing their new skills into their particular clinical setting [18, 21–23]. All enrolled mental health providers (regardless of randomization) will receive external facilitation. Facilitation is a dynamic, individualized process that typically bundles other implementation strategies [36]. For this project, external facilitation will include resolving implementation barriers, training and consultation, and technical support for the CALM tool. This study design feature will allow us to use an evidence-based strategy for implementing CBT while also determining whether CALM or manualized CBT results in improved fidelity to the treatment model. The external facilitator (an individual not located at the implementation site but who has knowledge and experience with VA practices and procedures) will help each provider develop an implementation plan, including setting individual goals for CBT implementation and helping to resolve barriers that emerge to implementing CALM (computer tool or manual). The facilitator will be present at the training sessions to meet the providers face-to-face. Thereafter, the facilitator will meet by phone with each therapist at least once a month for 3 months and be available on an “as-needed” basis for an additional 3-month period [21]. Monthly calls between the facilitator and mental health provider will differ from consultation by focusing on developing a plan to implement the CBT delivery method (e.g., how to use CALM or manual in session), generating solutions to barriers to CBT implementation (e.g., scheduling, time management), and maintaining motivation to practice CBT and the assigned delivery method.

### Training and consultation

All providers will receive training in CBT. The training will be of the same length and format (3-day, face-to-face) as initial training programs in VA rollouts of EBPs [37]. The training will differ across the two groups (those randomized to receive the CALM tool versus manual) in that the final 2 h of each day will involve practice with either delivery method. All providers who complete the training (regardless of randomization) will receive consultation for 3 months, which will consist of small group discussion with “audit and feedback” reports to therapists. Consultation will focus on coaching and giving providers feedback on the delivery of CBT. Both elements have been shown to be important in ensuring proficient delivery of EBPs [38]. Providers will audiotape all of their clinical sessions, and the consultant will review a sample of audiotapes from their sessions during the first 3 months. The consultant will review all recordings from the providers’ first patient and will provide written (via e-mail) or oral (via telephone) feedback after the second and last sessions. If a provider appears to need additional instruction, the supervisor will provide weekly individual consultation over the telephone for the provider until both agree that any problems have been resolved.

### Technical assistance for the CALM tool

Providers randomized to receive the CALM tool will receive ongoing technical support by phone. A technical support specialist will check in weekly with each provider using the CALM tool during the first month and will troubleshoot any problems that emerge. Thereafter, the technical support specialist will check in monthly but will also be “on call’ to address any technical challenges to using the CALM tool.

### Data collection

#### Fidelity assessments

Fidelity to the CBT model will be the primary outcome. Consistent with the recommendations [39], a multidimensional approach to measuring fidelity will be taken. We will use the fidelity rating scales employed in the prior CALM study in which fidelity is rated on a 1 to 7 scale using multiple items spanning three domains: protocol adherence, protocol competence, and global competence. Although separate ratings will be completed for each of these three domains, they will ultimately be combined to create a single measure of fidelity. We will add a fourth domain (non-adherence) developed just for this study. We believed this additional measure would be useful in identifying incidents of serious non-adherence to the CBT model which is associated with poorer treatment outcomes [40].

#### Fidelity rater selection, training, and procedures

Raters will be doctoral level CBT experts who either attend or review an audio recording of the training workshop for study therapists. They will also be provided with the treatment manual. To establish initial inter-rater reliability, each of two raters will independently rate an audiotape of a session and share his/her item-by-item ratings to determine a consensus rating for each item when discrepancies are found. This procedure will be repeated for a minimum of five sessions and until two consecutive sessions have a percentage of agreement on items of ≥80 % for the initial ratings.

#### Fidelity ratings

Fidelity assessments will focus on therapy sessions delivered early and late in the project. Fidelity for the first patient will be assessed and used to control for initial group differences in multivariate analyses. We expect each therapist to see ten patients each, with each patient completing up to eight sessions. Fidelity will be assessed only for patients who have attended at least four sessions. Fidelity will be measured for a “midpoint” patient (patient four or five of ten) and the last patient receiving at least four treatment sessions from each therapist. Fidelity for the midpoint and last patient for each therapist is our primary outcome measure. A single overall fidelity rating will be completed for each (midpoint and last) patient’s last three sessions. A minimum of 5 % of the total sessions being analyzed will be randomly selected to be rated by both raters to characterize inter-rater reliability.

#### Clinical outcomes

The clinical status of veterans participating in the study will be assessed at baseline, 3, and 6 months post randomization. The baseline assessment will be conducted prior to beginning CBT treatment. Assessments will be administered over the phone by a trained staff using a computer-assisted telephone interviewing system. The follow-up outcome assessments will include both generic measures applicable to all patients and disorder-specific measures.

### Data analysis

Providers will be the unit of the intent-to-treat analysis for measuring our primary (fidelity to the CBT model) and secondary (mental health) outcomes. For our primary outcomes, the dependent variable will be the composite fidelity score from each provider’s midpoint and last patient. The composite score will be calculated as the mean of the two scores. A dummy variable representing treatment group assignment will be specified as the explanatory variable of interest. An alpha significance level of 0.05 will be used to reject/accept the null hypothesis. Study hypotheses will be tested using a standard logistic regression model. Three covariates will be specified to control for differences across providers. We will first examine differences in the characteristics of providers across the intervention and control group and include those characteristics that differed statistically. If no group differences are observed in the characteristics of the providers, we will control for previous CBT experience (none, some, most experience) and length of time in clinical practice. To examine our secondary outcomes, overall mental health functioning will be measured at baseline and at 3 and 6 months follow-up. Patients will also be screened for any of five specific disorders at baseline (PTSD, PD, GAD, SAD, and comorbid depression); and for those who score positive in a given disorder, ongoing assessment of that disorder will continue through the study. We expect a high degree of comorbidity, so participants will choose the disorder that is bothering them the most as the “target” disorder for CBT treatment.

### Power analysis

The power calculation for our *primary outcome* takes into consideration analyses involving comparisons of means and proportions. In making these power calculations, we assume that two therapists in each group (four in total) will either “drop out” of the project, change jobs, or leave VA employment, leaving us with a sample size of 30. With a sample size of *n* = 30 therapists, we will have an 84 % power to detect a large effect size (Cohen’s *d* = 1.0) in mean treatment fidelity based on all sessions from each therapist’s mid and last patients. This calculation assumed a 1.2 point difference in mean fidelity between the intervention and control groups, a pooled standard deviation of 1.2, alpha = 0.5, and a two-tailed test of significance. With a sample size of *n* = 30 providers, we will also have 88 % power to detect a 35 % difference (85 versus 50 %) in the proportion of therapists maintaining minimally acceptable treatment fidelity between the intervention and control groups based on all sessions from each providers mid and last patients. This calculation assumed a 0 % attrition rate, alpha = 0.05, and a two-tailed test of significance.

It will not be necessary for patients to complete a full course (eight sessions) of CBT to be included in the study so we expect minimal “drop out” of patients. The power calculation for our *secondary outcome* must take into account the clustering of patients with providers. Prior psychotherapy RCTs have found intraclass correlation coefficients to vary widely (range 0 to 0.73) with a mean of about 0.08 [41]. We based our power analysis on a slightly conservative intraclass correlation coefficient of 0.10. With a sample size of *n* = 300, we will have 85 % power to detect a medium effect size (Cohen’s *d* = 0.5) for our secondary outcome of overall mental health functioning [42].

### Aim 3: implementation needs assessment

Our research partners in VA Central Office are interested in adopting the CALM tool for use in the VA, especially in CBOCs. If the CALM tool improves fidelity to the CBT model, our VA partners will help us pave the way for national rollout. To help prepare for the future implementation of the tool in routine care, we will perform an implementation needs assessment with key stakeholders—CBOC mental health providers participating in aim 2 and their CBOC directors.

### Study participants

All providers who participated in the effectiveness-implementation trial will be invited to take part in a focus group at the end of the trial. In addition, we will recruit a focus group of CBOC directors from sites that participated in the trial.

### Data collection

We will conduct focus groups with the mental health providers to assess their perspectives (i.e., what was helpful or not) about the CBT training, the use of the CALM tool (or not) in providing CBT, the consultation they received, and the additional facilitation and support provided to them and their CBOCs to use the computer program. In addition, we will ask them to comment on the experienced and expected barriers and facilitators to using the CALM tool in routine care. After the focus groups with mental health providers are completed, we will conduct a focus group with a sample of CBOC directors from participating sites, to assess their perspectives on the implementation of the CALM tool under routine care circumstances, and we will get their reaction to the barriers and facilitators raised by the mental health providers. The focus groups will take place on the telephone.

### Data analysis

The focus groups will be audiotaped, transcribed, and analyzed using “traditional content analysis” techniques which are largely inductive [43]. Two coders will code the qualitative data. Coding is analogous to developing a book index, with coders linking blocks of text to a code or group of codes [44, 45]. Coding is a major part of what Miles and Huberman [46] refer to as “data reduction” whereby “meaningful labels are attached to data chunks” (pp. 89). In general, we will note patterns that seem salient due to their recurring nature (both within and across interviews) or the extent of responses devoted to them. We will divide the data coding process into four steps:

1. Data management. We will enter verbatim interview transcripts into a qualitative data analysis software package that enables researchers to mark blocks of text with thematic codes and explore relationships among codes and between codes/participant groups.
2. Open coding. Two coders will review the interview guides and transcripts line-by-line and begin to identify key emerging themes. This open coding approach allows the discovery of themes that appear regularly, leading to development of initial “top-level” codes [47].
3. Top-level coding. After coming to consensus on the top-level codes (key themes and categories), the investigators will then apply these codes to the focus group transcripts (two to three transcripts each depending on the number of focus groups completed). To ensure coding reliability and consistency, both coders will review one another’s work and discuss and resolve differences.
4. Subcoding. After completing top-level coding, both coders will subcode top-level codes that are “grounded” (i.e., associated with repeated quotations/discussions), using the same methods described above for top-level coding. Subcoding entails further refinement of the broad constructs represented by the top-level codes into subcategories.

The final steps after coding are interpreting results and drawing conclusions [48]. As we have done in our previous research of this type, we will ultimately develop a large, segmented table with an accompanying narrative that lists, characterizes, and describes in detail the major barriers and facilitators of implementing the CALM tool (including the associated training, consultation, and facilitation necessary). This information will be subsequently used to develop an implementation toolkit to support widespread implementation in routine VA care.

### Study status

This study is currently underway. At the time of manuscript submission, we have recruited three (out of four) groups of stakeholders (mental health providers, CBT experts, and experts in the implementation of EBPs within VA) to participate in the first wave of focus groups described in aim 1.

## Discussion

This study will help determine whether a modified version of the CALM tool improves fidelity to CBT, compared to a manual version, in VA CBOCs. External facilitation will be used to implement both CBT delivery methods, which is needed given that the implementation of EBPs in smaller, more remote clinics is a substantial challenge. External facilitation has been shown to be a key strategy to promoting the adoption of EBPs both within and outside VA and will therefore be used as the overarching implementation strategy in the present study. In addition, this study will help support the VA mandate that mental health providers be prepared to treat a wide variety of mental health conditions of which the most common are anxiety disorders, PTSD, and depression. The CALM tool is specifically designed to help providers address these conditions and has the added benefit of teaching and refining providers’ CBT treatment skills as they proceed through the computer program. Furthermore, this project will provide important information about the clinical effectiveness of the CALM tool for veterans with these mental health conditions and provide qualitative data on how to optimize the implementation of CBT, and specifically CALM within VA CBOCs. This is important given that mental health providers in VA CBOCs are often isolated from their colleagues and may have few educational resources that may help master EBPs. If successful, this study may provide useful data that could help inform a nationwide implementation of the CALM tool in VA CBOCs to help support mental providers in delivering CBT and improve patient outcomes.
